# Blue-light only: How horsetails broke the rules of stomatal control

**DOI:** 10.1093/plphys/kiaf298

**Published:** 2025-07-03

**Authors:** Ritu Singh, Erin Cullen

**Affiliations:** Assistant Features Editor, Plant Physiology, American Society of Plant Biologists; Department of Plant Science, University of California, Davis, CA 95616, USA; Assistant Features Editor, Plant Physiology, American Society of Plant Biologists

Seed plants display highly responsive stomatal behavior, modulated by environmental cues such as light, humidity, and CO₂ concentration. One hallmark adaptation is their stomatal response to low-intensity blue light in the presence of red light, allowing plants to maximize stomatal aperture independently of direct photosynthetic demand (Vialet-Chabrand et al. 2021). While this mechanism is well characterized in angiosperms, many seedless vascular plants have been considered unresponsive to such light cues (McAdam and Brodribb 2012). However, some aquatic or sun-adapted fern lineages, like the Marsileaceae family, have evolved unusually fast and responsive stomatal systems (Westbrook and McAdam 2021), hinting at an underappreciated diversity of stomatal behavior in the pteridophyte lineage.

One such exception is the ancient genus *Equisetum*, commonly known as horsetails. These spore-producing vascular plants thrive in sunny, water-saturated environments and maintain gas exchange rates comparable to those of many angiosperms (Husby et al. 2014). *Equisetum* has a striking overall morphology characterized by silica-rich stems and a fossil lineage dating back to the Jurassic. The stomata of *Equisetum* are also unique, with 2 subsidiary cells above the guard cells forming the stomatal pore. In addition, mature *Equisetum* stomata exhibit prominent silicified thickenings on the lower wall of subsidiary cells (Cullen and Rudall 2016). However, how *Equisetum* regulates its stomata opening and closing has remained unclear. A recent study by McAdam et al. (2025), published in *Plant Physiology*, addresses this gap by focusing on *Equisetum praealtum* (also known as *E. hyemale* subsp. *affine*), a widespread evergreen species found in both temperate and boreal zones across North America and East Asia. Frequently considered a nuisance weed in agriculture, *E. praealtum* grows in full sun with roots anchored in consistently wet soils (Rutz and Farrar 1984).

Using infrared gas analysis, the researchers monitored diurnal stomatal conductance in *E. praealtum* under natural sunlight. They observed rapid stomatal opening at dawn and equally swift closure at dusk, a pattern far more responsive than that observed in most other pteridophytes. The strong alignment between photosynthetically active radiation and stomatal conductance throughout the day suggests that *Equisetum* has evolved a tightly light-coupled gas exchange strategy. Additionally, using potted plants in a glasshouse, the authors tracked canopy conductance across 4 consecutive sunny days and found a strong linear correlation with declining light intensity during the evening, reinforcing the strong coupling between light availability and stomatal behavior. The strong coordination between light intensity and plant gas exchange, driven by very rapid stomatal closure as light levels decline, might allow *Equisetum* to greatly reduce gas exchange at night; however, further experiments are required to investigate this hypothesis.

To investigate the light specificity of this response, the authors exposed plants to precisely controlled red and blue light treatments. They found that both *E. praealtum* and its relative *E. diffusum* exhibited robust stomatal opening under blue light alone but showed no stomatal response to red light, even at high intensities. Upon removal of blue light, either by switching to darkness or maintaining only red light, stomata closed rapidly (Figure). This complete dependence on blue light, with no red light responsiveness, is rare among vascular plants. While most seed plants exhibit a synergistic blue-red light response, and even orchids with partial red insensitivity retain some red-light responsiveness, *Equisetum* appears to have an exclusive stomatal reliance on blue light.

**Figure.**
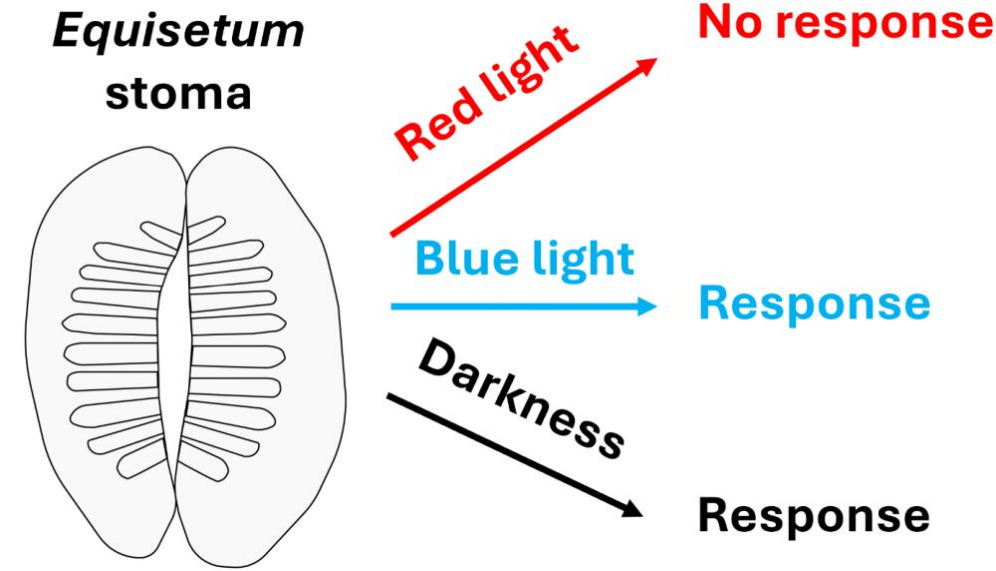
The unique stomata of *Equisetum* respond to blue light and darkness, but not red light. A paradermal section through a mature *Equisetum* stomatal complex is illustrated, radiating silica ridges on the subsidiary cells are visible. Based on Cullen and Rudall (2016).

Why has *Equisetum* evolved this distinctive regulatory mechanism? The authors offer 2 hypotheses. First, ecologically, blue light is a dominant component of full-sunlight environments, providing a consistent and reliable signal for gas exchange. In such habitats, blue-light–driven stomatal opening may optimize resource use by aligning gas exchange with peak photosynthetic opportunity. Second, the blue-light-only mechanism may serve to uncouple stomatal behavior from photosynthetic feedback. For a wetland-adapted species like *Equisetum*, where water conservation is less critical, this decoupling could allow maximum CO₂ uptake in their specialized stem-based photosynthetic tissues.

Anatomical evidence may provide further insights. *Equisetum* guard cells lack chlorophyll-containing plastids (Cullen and Rudall 2016), an unusual trait shared with only a few plant groups, such as some orchids (Nelson and Mayo 1975). In seed plants, red-light responses often rely on chloroplast-mediated pathways, so the absence of plastids may explain the lack of red-light–induced stomatal opening in *Equisetum*. However, the alignment between plastid loss and blue-light exclusivity may also point to deeper mechanistic innovations, perhaps involving unique ion channels or membrane properties in guard cells, that remain to be explored. In the future, given the unusual stomatal morphology of *Equisetum*, it would be interesting to test the link between form and function. For example, does stomatal regulation alter depending on the age of the plant?

The implications of this work extend beyond the biology of *Equisetum*. It challenges the long-held assumption that stomatal behavior is broadly conserved across vascular plants. Although core developmental genes involved in stomatal formation may be shared, this study demonstrates that relatively minor modifications in signaling pathways or regulatory mechanisms could yield intriguing physiological diversity. It also underscores the limitations of extrapolating from model organisms such as *Arabidopsis* to more distantly related groups.

More broadly, this work contributes to a growing recognition that ancient lineages are capable of remarkable physiological innovation. From circadian-regulated stomata in Marsileaceae to hypersensitive blue-light–driven stomatal control in *Equisetum*, pteridophytes are rewriting our understanding of plant gas exchange. As the impacts of climate change intensify, insights into diverse stomatal strategies across lineages will be critical for predicting and improving plant responses to changing environments. In this context, *Equisetum* serves as a powerful reminder that ancient lineages can evolve modern solutions.

## Data Availability

No data were generated or analyzed in this study.
